# Geographic shift of Japanese spotted fever and diagnostic overshadowing by COVID-19 testing: A genetically confirmed northernmost case

**DOI:** 10.1016/j.idcr.2025.e02361

**Published:** 2025-09-12

**Authors:** Hiroaki Hara, Jiro Kamiyama, Ryoichiro Kondo, Shintaro Furuya, Shigemasa Taguchi, Yasushi Shibue, Motohiko Ogawa, Yuki Hashimoto, Koji Kikuchi, Kazuya Kiyota

**Affiliations:** a Saitama Red Cross Hospital Advanced Emergency Medical Center, Saitama City, Japan; b Yokohama City Minato Red Cross Hospital Department of Infectious Diseases, Yokohama City, Japan; c National Institute of Infectious Diseases, Shinjuku City, Tokyo, Japan; d Saitama City Institute of Health Science and Research, Saitama city, Saitama, Japan

**Keywords:** COVID-19, Japanese spotted fever, Tetracycline, Rickettsial disease, Sepsis

## Abstract

Japanese spotted fever (JSF), caused by Rickettsia japonica, typically presents with fever, rash, and eschar. While endemic to western and southern Japan, recent cases have emerged in urban, non-endemic areas. A 49-year-old man developed fever after returning from a work assignment in Yamagata Prefecture, a historically non-endemic region. Despite no respiratory findings, he tested positive for SARS-CoV-2 antigen and was initially treated as COVID-19. His condition deteriorated into multiorgan dysfunction, requiring intensive care. Physical examination revealed a diffuse rash and an eschar on the lower leg. Rickettsial infection was suspected, and intravenous minocycline was initiated. Rickettsia japonica was later confirmed by PCR and serology. This case represents the northernmost genetically confirmed JSF in Japan and emphasizes the importance of maintaining a broad differential diagnosis, particularly when COVID-19 test results conflict with clinical findings.

## Graphical Abstract

**Figure:**
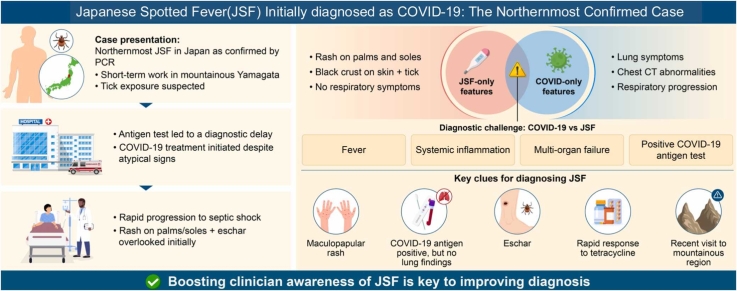

## Introduction

Japanese spotted fever (JSF) is a tick-borne disease caused by *Rickettsia japonica*, first reported in 1984. It typically presents with high fever, diffuse maculopapular rash, and a necrotic eschar at the tick bite site—clinical features that are considered hallmark findings of the disease. JSF has traditionally been reported in rural, mountainous regions of western and the southern Kanto region. However, over the past decade, the annual incidence has tripled, and cases have emerged in previously non-endemic areas, including urban settings, indicating geographic expansion. If unrecognized, JSF may progress to severe systemic illness with multiorgan failure. Early treatment with tetracyclines, such as doxycycline or minocycline, is associated with markedly improved outcomes.

During the COVID-19 pandemic, patients presenting with fever and shock are often presumed to have SARS-CoV-2 infection or related inflammatory syndromes. Although COVID-19 can mimic systemic infections, pulmonary involvement is typically prominent. When respiratory findings are absent, alternative diagnoses such as rickettsial illness should be carefully considered.

We present a case of severe JSF initially misdiagnosed as COVID-19 in an urban emergency department, highlighting the importance of thorough physical examination and early targeted treatment in suspected rickettsial disease.

## Case report

### Initial presentation and diagnosis

In early November 2023 (Day 0), a 49-year-old previously healthy man developed fever and malaise after returning home to Saitama Prefecture—an urban area just north of Tokyo—following a short-term work assignment in a mountainous region of Yamagata Prefecture in northern Japan. He initially presented to a local hospital in Saitama with these symptoms on the third day after symptom onset (Day 3). Despite the absence of respiratory findings on imaging and physical examination, he tested positive for SARS-CoV-2 on a rapid antigen test and was treated as an outpatient with ensitrelvir fumaric acid and loxoprofen sodium. However, his epigastric pain newly appeared over the following 2 d (Day 5), prompting presentation to the emergency department of a different hospital, where he was admitted and started on ceftriaxone. As his condition rapidly deteriorated and progressed to multiorgan dysfunction, diagnostic uncertainty emerged. On Day 5 (same day of second presentation), he was urgently transferred to our tertiary care center with a working diagnosis of multisystem inflammatory syndrome in adults (MIS-A), a post-infectious complication increasingly recognized in the context of COVID-19[1].

### Critical care course

On arrival, he had slight fever (37.1 ℃), hypotensive, and tachypneic. Laboratory tests showed marked systemic inflammation, thrombocytopenia, hyperbilirubinemia, acute kidney injury and coagulopathy. He required vasopressor support, mechanical ventilation, and continuous renal replacement therapy (CRRT). Relevant laboratory data are presented in Table 1.

**Table 1:** Laboratory findings at presentation. Laboratory values at admission showing inflammation, hepatic and renal dysfunction, and coagulopathy.

**Electrolytes and Metabolites**		
Test	Result	Unit
Sodium	128	mEq/L
Potassium	4.00	mEq/L
Chloride	93.0	mEq/L
Calcium	7.10	mg/dL
Phosphate	5.20	mg/dL
Magnesium	1.70	mg/dL
Albumin	4.80	g/dL
Total Bilirubin	2.40	mg/dL
Blood Urea Nitrogen	72.4	mg/dL
Creatinine	5.17	mg/dL
Aspartate Aminotransferase	154	IU/L
Alanine Aminotransferase	100	IU/L
Alkaline Phosphatase	107	IU/L
Gamma-Glutamyl Transferase	29.0	IU/L
Creatine Kinase	179	IU/L
C-reactive Protein	33.2	mg/dL
Ferritin	9200	ng/mL
Procalcitonin	4.71	ng/mL
B-type Natriuretic Peptide	29.3	pg/mL
**Hematology**		
Test	Result	Unit
White Blood Cell Count	14000	/μL
Segmented Neutrophils	95.0	%
Stab Neutrophils	0	%
Lymphocytes	3.2	%
Eosinophils	0	%
Basophils	0.7	%
Monocytes	1.1	%
Hemoglobin	13.1	g/dL
Hematocrit	35.9	%
Platelet Count	45000	/μL
**Coagulation Tests**		
Test	Result	Unit
Prothrombin Time	74.0	%
Activated Partial Thromboplastin Time	39.7	sec
Fibrinogen	347	mg/dL
Antithrombin III	68.0	%
Fibrin Degradation Products	25.0	μg/mL
**Blood Gas Analysis**		
Test	Result	Unit
pH	7.44	
Partial Pressure of CO₂ (PCO₂)	27.2	Torr
Partial Pressure of O₂ (PO₂)	85.1	Torr
Bicarbonate (HCO₃⁻)	18.1	mEq/L
Base Excess	−4.5	
Lactate	2.29	mmol/L

**Fig. 1:**
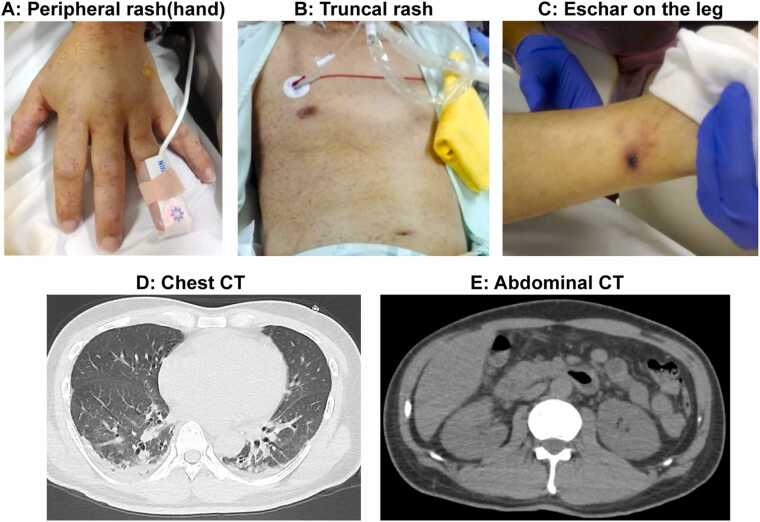
Clinical photographs. A: Peripheral rash (hand). B: Diffuse truncal rash. C: Eschar on the lower leg. D: Chest CT. E: Abdominal CT.

Physical examination revealed a 5-mm black eschar on the left posterior aspect of the left lower leg and a diffuse maculopapular rash over the trunk and extremities, including the palms and soles (Fig. 1). Intravenous minocycline (200 mg/d), which is effective against both scrub typhus and JSF, was initiated on hospital day 1in consideration of suspected rickettsial infection. Given the patient’s recent stay in the Tohoku region—where scrub typhus is historically more prevalent—scrub typhus was initially considered the most likely diagnosis.

Soon after admission, worsening metabolic acidosis (pH 7.20) prompted endotracheal intubation and mechanical ventilation. Despite norepinephrine infusion at 0.4 μg/kg/min, systolic blood pressure remained between 50 and 60 mmHg. Crystalloids were infused at 300 mL/h, and vasopressin (2 U/h) and hydrocortisone (200 mg/d) were added. Transthoracic echocardiography revealed diffuse left ventricular hypokinesis with a severely reduced ejection fraction of 20–30 %, prompting initiation of dobutamine at 2 μg/kg/min. CRRT was commenced to manage refractory metabolic acidosis. During the first 48 h, vasopressor requirements increased, and the Sequential Organ Failure Assessment (SOFA) score reached a peak of 19 on day 1. With intensive supportive care, the patient steadily improved: vasopressors were weaned off by day 5, CRRT discontinued by day 6, and he was extubated on day 11. Inflammatory markers and organ function gradually normalized.

On hospital day 14, *Rickettsia japonica* was identified using PCR from the eschar, and paired serology showed a fourfold rise in IgM and IgG titers. For detailed microbiologic findings (refer to Fig. 3).

**Fig. 2:**
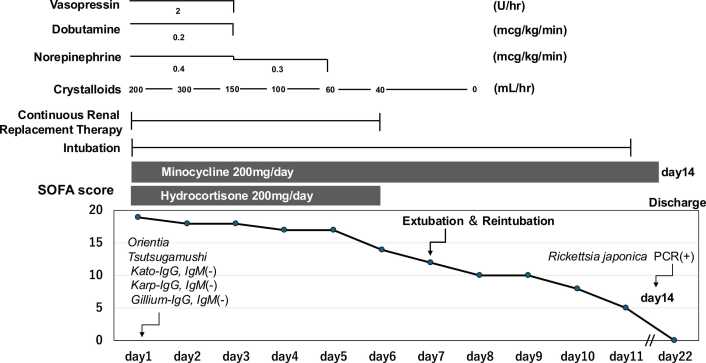
**SOFA score progression.** Sequential Organ Failure Assessment (SOFA) score from Day 1 to Day 22, illustrating the association between minocycline initiation and organ function recovery.

The SOFA score trajectory is depicted in Fig. 2, clearly illustrating the temporal association between therapeutic interventions—including minocycline administration and organ support—and subsequent organ function recovery beginning on day 3.

## Discussion

**Fig. 3:**
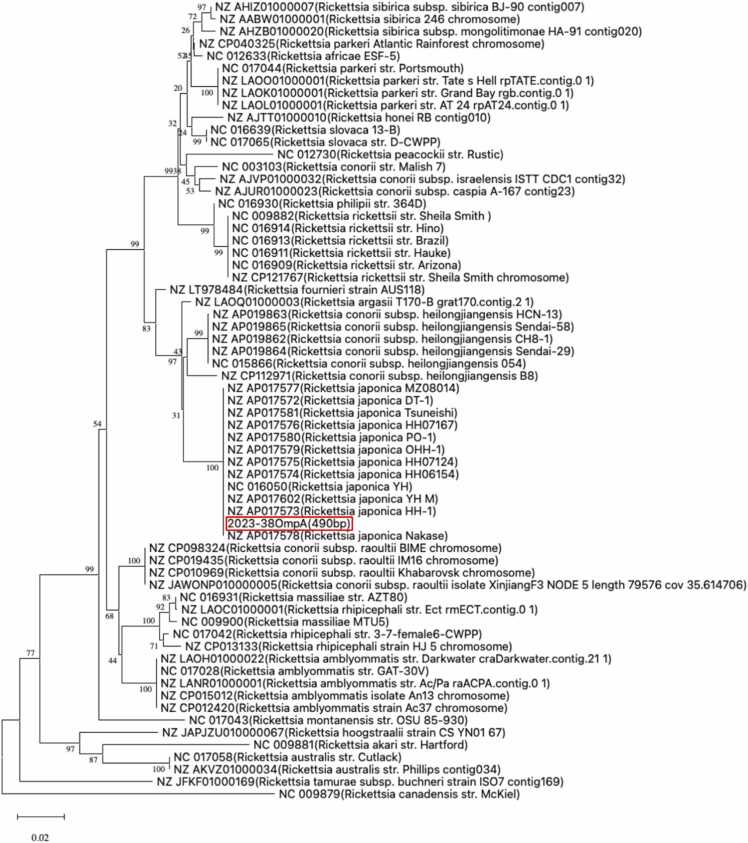
Phylogenetic analysis of the detected the spotted fever group rickettsia. The *omp*A gene sequence of the detected spotted fever group rickettsia is 100 % identical to this case. The *omp*A gene is discriminative among strains and confirmed the identification of the isolate as *Rickettsia japonica*.

This case highlights the diagnostic complexity of evaluating febrile illness during the COVID-19 pandemic, particularly when a positive SARS-CoV-2 antigen result prematurely narrows diagnostic considerations [2]. Although SARS-CoV-2 infection and JSF can manifest with systemic inflammation, coagulopathy, and multiorgan dysfunction, key clinical features facilitate differentiation. In this patient, the absence of respiratory symptoms and normal chest imaging were inconsistent with COVID-19. In contrast, the constellation of findings—including a diffuse maculopapular rash involving the palms and soles, a small necrotic eschar, and distributive shock—was more characteristic of a rickettsial illness Fig. 3.

Gastrointestinal symptoms such as abdominal pain are reported in both JSF and COVID-19 [3]. However, in JSF, they occur in up to 40 % of cases and are believed to reflect hepatic inflammation or vasculitis involving the gastrointestinal tract [4]. In contrast, abdominal pain in the absence of respiratory symptoms is relatively uncommon in COVID-19. In this case, epigastric pain was the earliest symptom and contributed to initial diagnostic ambiguity, particularly in the context of a positive antigen test. Nonetheless, the prompt clinical response to presumptive tetracycline therapy, along with confirmatory microbiologic testing, ultimately established the diagnosis of JSF.

This case also underscores the hazards of diagnostic anchoring during a global pandemic. Although COVID-19 understandably dominates clinical attention, it is imperative that clinicians maintain a broad differential diagnosis—especially in patients with atypical presentations or discordant clinical findings. Thorough physical examination, including meticulous skin inspection was essential to reaching the correct diagnosis, particularly in a region where JSF is rarely encountered and often overshadowed by scrub typhus in the differential diagnosis.

**Fig. 4:**
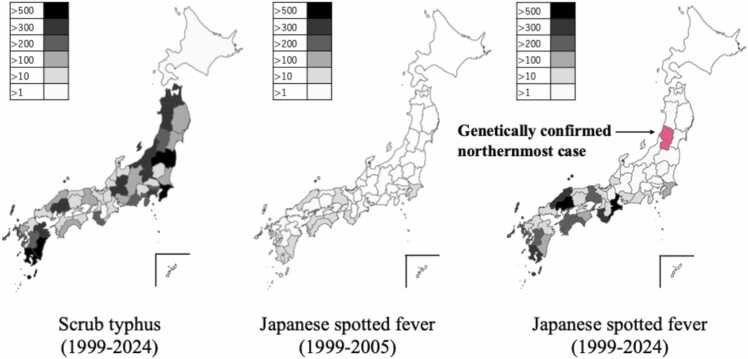
Cumulative reported cases of Japanese spotted fever and Scrub typhus in Japan from 1999–2024 [7]. Data from the National Institute of Infectious Diseases (NIID) demonstrate the geographic expansion of JSF into more northerly regions in recent years.

Since 2023, the annual reported cases of JSF have surpassed those of scrub typhus in Japan, reflecting a progressive expansion into northern and urban areas [5]. This trend is supported by national surveillance data from 2001–2020, which demonstrate a nearly tenfold increase in annual incidence rates (average annual percent change: +12.3 %), with steady growth in cooler regions such as the Tohoku and Kanto areas [6]. As illustrated in Fig. 4, the geographic range of JSF has progressively expanded from its historical foci in western and southern Japan toward northern regions over the past two decades.

Historically, however, JSF has been exceedingly rare in the Tohoku region, where scrub typhus has long been considered the predominant rickettsial disease and prioritized in the differential diagnosis of febrile illnesses with eschar. Although JSF can be reported based solely on serology, such cases in the Tohoku region have not been confirmed by genetic detection and strain typing. In contrast, this case was confirmed by both PCR and serology, including genetic identification of the strain, represents the northernmost genetically proven JSF infection to date, extending the recognized distribution range of the disease and underscoring the importance of considering JSF in the differential diagnosis even in regions where it was previously considered rare.

This case reinforces the need for early presumptive tetracycline therapy and underscores the importance of continuously re-evaluating diagnostic assumptions—even during pandemics—to prevent potentially fatal delays in the treatment of rickettsial infections.

## Conclusion

JSF should remain a key consideration in febrile patients with rash and systemic illness, even in urban or non-endemic areas. This case highlights the importance of careful physical examination and early treatment with tetracyclines when rickettsial infection is suspected—particularly when COVID-19 test results are discordant with clinical findings.

## Author statement

We sincerely thank you for the valuable comments on our manuscript (IDCR-D-25–00411). In response, we have revised the manuscript accordingly. The main changes include clarifying the timeline between illness onset and hospital admission, removing sedative medications from Fig. 2, and adding regional epidemiologic data and a map to the Discussion.

We believe these revisions have improved the clarity and scientific value of our manuscript. We appreciate your consideration.

## Author agreement

All authors certify that they have made substantial contributions to the conception, design, data acquisition, analysis, or interpretation of data; have drafted or critically revised the manuscript for important intellectual content; and have approved the final version of the manuscript. The manuscript is original, has not been published elsewhere, and is not under consideration by another journal. All authors agree to be accountable for all aspects of the work. Written informed consent was obtained from the patient for publication of this case report and any accompanying images, as described below. No funding was received for this work. The data supporting the findings of this study were obtained from the National Institute of Infectious Diseases and are available from the corresponding author upon reasonable request.

## CRediT authorship contribution statement

**Yuki Hashimoto:** Resources. **Koji Kikuchi:** Resources. **Yasushi Shibue:** Writing – review & editing. **Motohiko Ogawa:** Resources. **Shigemasa Taguchi:** Supervision. **Kazuya Kiyota:** Supervision. **Ryoichiro Kondo:** Resources. **Shintaro Furuya:** Resources. **Hiroaki Hara:** Writing – review & editing, Writing – original draft, Project administration, Data curation, Conceptualization. **Jiro Kamiyama:** Writing – review & editing.

## Consent for publication

Written informed consent was obtained from the patient for publication of this case report and any accompanying images.

## Ethics approval and consent to participate

Not applicable.

## Funding

The research did not receive any specific grant from funding agencies in the public, commercial, or not-for-profit sectors.

## Declaration of Competing Interest

The authors declare that they have no known competing financial interests or personal relationships that could have appeared to influence the work reported in this paper.

## Data Availability

The data supporting the findings of this study were obtained from the National Institute of Infectious Diseases and are available from the corresponding author upon reasonable request.
